# Exploring risk factors for autoimmune diseases complicated by non-hodgkin lymphoma through regulatory T cell immune-related traits: a Mendelian randomization study

**DOI:** 10.3389/fimmu.2024.1374938

**Published:** 2024-05-28

**Authors:** Qi Liu, Xintong Zhou, Kunjing Liu, Yimin Wang, Cun Liu, Chundi Gao, Qingqing Cai, Changgang Sun

**Affiliations:** ^1^ The First Clinical Medical College, Shandong University of Traditional Chinese Medicine, Jinan, China; ^2^ School of Traditional Chinese Medicine Department, Beijing University of Chinese Medicine, Beijing, China; ^3^ College of Traditional Chinese Medicine, Shandong Second Medical University, Weifang, China; ^4^ Department of Medical Oncology, Sun Yat-sen University Cancer Center, Guangzhou, China; ^5^ State Key Laboratory of Oncology in South China, Guangdong Provincial Clinical Research Center for Cancer, Collaborative Innovation Center of Cancer Medicine, Sun Yat-sen University Cancer Center, Guangzhou, China; ^6^ Department of Oncology, Weifang Traditional Chinese Hospital, Weifang, China

**Keywords:** regulatory T cell, autoimmune disease, non-Hodgkin lymphoma, Mendelian randomization, GWAS data

## Abstract

**Background:**

The effect of immune cells on autoimmune diseases (ADs) complicated by non-Hodgkin lymphoma (NHL) has been widely recognized, but a causal relationship between regulatory T cell (Treg) immune traits and ADs complicated by NHL remains debated.

**Methods:**

Aggregate data for 84 Treg-related immune traits were downloaded from the Genome-Wide Association Study (GWAS) catalog, and GWAS data for diffuse large B-cell lymphoma (DLBCL; n=315243), follicular lymphoma (FL; n=325831), sjögren’s syndrome (SS; n=402090), rheumatoid arthritis (RA; n=276465), dermatopolymyositis (DM; n=311640), psoriasis (n=407876), atopic dermatitis (AD; n=382254), ulcerative colitis (UC; n=411317), crohn’s disease(CD; n=411973) and systemic lupus erythematosus (SLE; n=307587) were downloaded from the FinnGen database. The inverse variance weighting (IVW) method was mainly used to infer any causal association between Treg-related immune traits and DLBCL, FL, SS, DM, RA, Psoriasis, AD, UC, CD and SLE, supplemented by MR-Egger, weighted median, simple mode, and weighted mode. Moreover, we performed sensitivity analyses to assess the validity of the causal relationships.

**Results:**

There was a potential genetic predisposition association identified between CD39+ CD8br AC, CD39+ CD8br % T cell, and the risk of DLBCL (OR=1.51, p<0.001; OR=1.25, p=0.001) (adjusted FDR<0.1). Genetic prediction revealed potential associations between CD25++ CD8br AC, CD28- CD25++ CD8br % T cell, CD39+ CD8br % CD8br, and the risk of FL (OR=1.13, p=0.022; OR=1.28, p=0.042; OR=0.90, p=0.016) (adjusted FDR>0.1). Furthermore, SLE and CD exhibited a genetically predicted potential association with the CD39+ CD8+ Tregs subset. SS and DM were possibly associated with an increase in the quantity of the CD4+ Tregs subset; RA may have reduced the quantity of the CD39+ CD8+ Tregs subset, although no causal relationship was identified. Sensitivity analyses supported the robustness of our findings.

**Conclusions:**

There existed a genetically predicted potential association between the CD39+ CD8+ Tregs subset and the risk of DLBCL, while SLE and CD were genetically predicted to be potentially associated with the CD39+ CD8+ Tregs subset. The CD39+ CD8+ Tregs subset potentially aided in the clinical diagnosis and treatment of SLE or CD complicated by DLBCL.

## Introduction

Non-Hodgkin’s lymphoma (NHL) is a malignant tumor of the blood system originating from lymph nodes and other extranodal lymphoid tissues, with significant heterogeneity. Its onset is closely associated with autoimmune diseases (ADs), especially systemic lupus erythematosus (SLE), rheumatoid arthritis (RA), and Sjogren’s syndrome (SS) (1–3). Klein et al. found that the risk of NHL in SLE patients was 4 - 7 times higher than that in the general population, especially for diffuse large B-cell lymphoma (DLBCL) and follicular lymphoma (FL) (4). Furthermore, compared to the general population, SS patients are at a higher risk of developing NHL, especially mucosa-associated lymphoid tissue (MALT) lymphoma, DLBCL, or lymphoplasmacytic lymphoma (5, 6). However, the mechanisms of ADs complicated by NHL still need further investigation.

Existing data show that regulatory T cells (Tregs; a subset of immunosuppressive T cells) effectively inhibit peripheral effector T cells by producing immunosuppressive cytokines, which play a key role in the pathogenesis of NHL and ADs. Multiple studies have shown that Tregs are highly expressed in B-cell type NHL, such as FL or DLBCL, and exhibit immunosuppressive function (7). This may be associated with the elevated expression levels of the surface receptors CTLA-4 and GITR on Tregs in the affected lymphoid tissues. In addition, malignant B cells in NHL promote the differentiation of T cells into Tregs by secreting TGF-β, while inhibiting effector T cells, further inducing the formation of an immune-suppressive tumor microenvironment. The absence or dysfunction of Tregs is found in various ADs, leading to a decrease in peripheral immune tolerance and promoting the occurrence and development of ADs. Observational studies have shown that the proportion of CD4+ CD25hi Tregs in the peripheral blood of patients with active SLE is reduced, and the proliferation and cytokine secretion of CD4+ effector T cells are poorly inhibited *in vitro (* 8, 9). Contradictory to this, Tregs increase in autoimmune and inflammatory diseases, but cannot effectively regulate the diseases. A high proportion of Foxp3+ Tregs expressing Helios are found in the circulating CD3+ population in cases of active SLE, and the number of these cells positively correlates with disease activity (10, 11). Elevated levels of cytokines or activated effector T cells stimulate compensatory immune responses in the body by increasing Tregs to exert immune suppression and maintain immune homeostasis. However, insufficient infiltration of Tregs makes it difficult to effectively suppress inflammatory signaling pathways, thus failing to achieve effective regulation of ADs (12, 13). After Toll receptor stimulation, dendritic cells secrete inhibitory cytokines that can suppress the T cell activation process mediated by Tregs; studies also find that IL-6 might play a key role in overcoming Tregs-mediated immune suppression (14). However, few studies have addressed the key role of Tregs in ADs complicated by NHL. Although some observational studies have attempted to explain the association between Tregs and ADs and NHL, the results may have been biased by unexpected confounding variables or reverse causality, making it difficult to establish a clear causal relationship.

MR can validate ADs and NHL relationship, identify genetic causality, and inform diagnostic and treatment strategies. Mendelian randomization (MR) analysis is based on the principle of random allocation of parent alleles to offspring following the Mendelian laws of inheritance and is used to assess causal relationships between exposure and outcome. It can be regarded as a “natural” randomized controlled trial (RCT) method, which can reduce the traditional bias of observational studies and make up for the shortcomings of RCTs in the study of rare diseases (15). However, MR has not been used to study any causal relationships between Tregs and ADs complicated by NHL. This study used single nucleotide polymorphism (SNP) data from a large whole-gene association study (GWAS) on immune-related traits of Tregs to reveal any causal relationships between immune-related traits of Tregs and ADs complicated by NHL through MR analysis. The aim was to provide strategies for the predictive diagnosis and treatment of ADs complicated by NHL.

## Methods

### Exposure and outcome data sources

Exposure and outcome sample data were obtained from different databases to avoid bias caused by overlapping exposure and outcome samples. Data on DLBCL (including 1050 cases and 314193 controls), FL (including 1181 cases and 324650 controls), RA (including 13621 cases and 262844 controls), Dermatopolymyositis (DM) (including 430 cases and 311210 controls), SS (including 2735 cases and 399355 controls), psoriasis (including 10312 cases and 397564 controls), atopic dermatitis (AD) (including 15208 cases and 367046 controls), ulcerative Colitis (UC) (including 5931 cases and 405386 controls), crohn’s disease (CD) (including 2033 cases and 409940 controls) and SLE (including 1083 cases and 306504 controls) were retrieved from the FinnGen public data source, which involved samples of European descent (**Supplementary Table S1**). The immune cell-related characteristic data was derived from the SardiNIA project, which analyzed 539 immune features using flow cytometry, evaluating a total of 731 cell features in 3757 individuals from the Sardinian population. The different subsets and functional states of Tregs were characterized by the surface markers CD3, CD4, CD8, CD25, CD127, CD45RA, CD28, and CD39. Summary statistical data on 84 Treg immune phenotypes were extracted, including absolute cell counts (AC; n=28) and relative cell counts (RC; n=56) (**Supplementary Table S2**) (16).

### Screening of instrumental variables

Considering the limited number of SNPs related to certain Tregs-related immune traits, a broad threshold (p<1e-5) was selected to extract instrumental variables (IVs) after consulting relevant literature (17). To ensure an adequate number of IVs to maintain statistical power, SNPs significantly associated with SLE, DLBCL, FL, SS, DM, CD (p<5e-6), and RA, AD, Psoriasis, UC(p<5e-8) were chosen as IVs. To mitigate the impact of linkage disequilibrium on the results, IVs for Tregs-related immune traits were pruned using a threshold of kb=10000, R^2^ = 0.001 (18), while DLBCL, FL, SLE, SS, DM, CD used kb=1000, R^2^ = 0.1, and RA, AD, psoriasis, UC used kb=1000, R^2^ = 0.01, with a reference panel of 1000 genomes from the European population (19). Palindromic SNPs were removed from the IVs. Additionally, to avoid bias from weak IVs, the F-statistic for each SNP was calculated to quantify the strength of IVs, and weak IVs (F<10) were excluded (20). A total of strong IVs related to Tregs-related immune traits (ranging from 8 to 691), 22 strongly associated with DLBCL, 72 with FL, 77 with SLE, 59 with RA, 134 with SS, 70 with AD, 99 with CD, 78 with Psoriasis, 66 with UC and 21 with DM were included.

### Two-sample MR analysis

**Figure 1:**
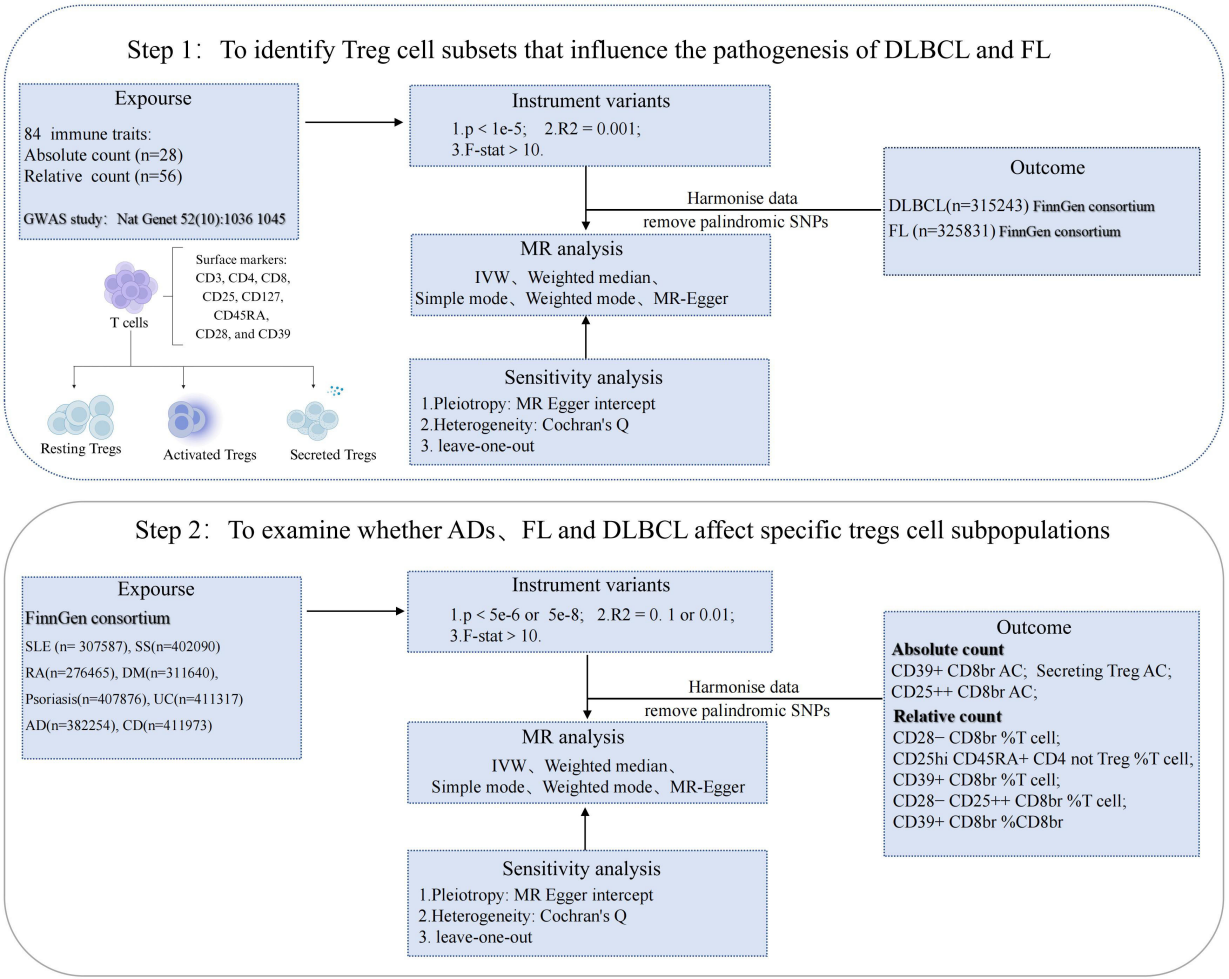
Study design and workflow: Key analysis MR Flow diagram. SLE, systemic lupus erythematosus; DLBCL, diffuse large B-cell lymphoma; FL, follicular lymphoma; GWAS, Genome-Wide Association Study; IVW, inverse variance weighted; ADs, autoimmune diseases; SS, sjögren's syndrome; RA, rheumatoid arthritis; DM, dermatopolymyositis; UC,ulcerative colitis; AD,atopic dermatitis; CD, crohn’s disease.

The theoretical basis for two-sample MR studies relies on three core assumptions (1): the selected genetic variants as instrumental variables are associated with risk factors (2); the selected genetic variants are significantly associated with outcome factors only through risk factors (3); the selected instrumental variables are not associated with other confounding factors (21). In MR analysis, inverse variance weighting (IVW) can improve the statistical power and accuracy of the estimates when core assumptions are satisfied. Therefore, based on the IVW method, the causal relationship between Tregs-related immune traits and DLBCL and FL, as well as between SLE, SS, RA, DM, psoriasis, AD, UC, CD and Tregs-related immune traits, was examined. To ensure the robustness of the results, MR-Egger, weighted median, simple mode, and weighted mode can be used as auxiliary methods. We used MR-Egger regression and IVW analysis to identify heterogeneity, which was measured by calculating Cochran’s Q. p values > 0.05 indicated that the heterogeneity was not significant. For p values < 0.05 (indicating heterogeneity), we used the random-effects model to reduce the accuracy of the estimates and address the problem of heterogeneity. Using the MR-Egger intercept test, the pleiotropy of the data can be detected and the robustness of the results evaluated. P values > 0.05 indicated the absence of pleiotropy. Leave-one-out analysis was used to detect whether the causal relationship between exposure and outcome was influenced by a single SNP. At the same time, in order to verify whether the research results are affected by multiple tests, the study also uses Q-value program to correct the False Discovery Rate (FDR) when the q value of FDR is < 0.1, a significant association is indicated (22). When p < 0.05 but q ≥ 0.1, exposure and outcome factors were considered suggestive (Study design and workflow, **Figure 1**).

## Results

### Estimation of a causal relationship between Treg immune characteristics and NHL types

**Figure 2:**
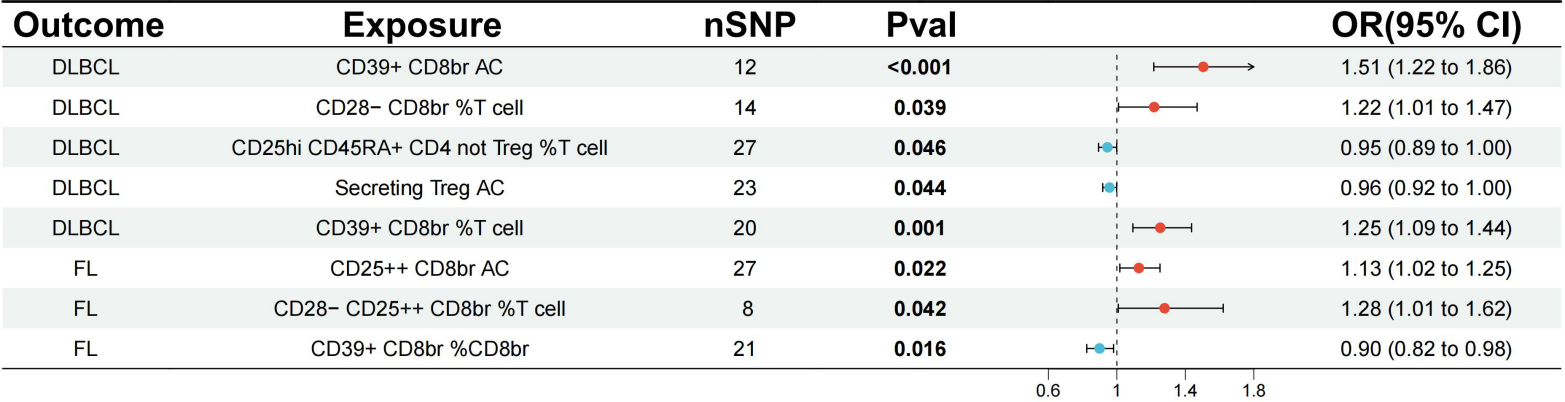
Evaluating the causal relationship between regulatory T cell immune-related traits and NHL-related risk using the IVW analysis method. nSNP, the number of single nucleotide polymorphisms; IVW, Inverse Variance Weighted; DLBCL, diffuse large B-cell lymphoma; FL, follicular lymphoma.

The study analyzed the correlation between 84 Tregs immune-related traits and DLBCL. Through IVW analysis, the results demonstrated a positive correlation between the CD39+ Tregs subset and the risk of DLBCL. Notably, CD39+ CD8br AC (OR=1.51, 95% CI=1.22-1.86, p<0.001, FDR=0.015) and CD39+ CD8br % T cell (OR=1.25, 95% CI=1.09-1.44, p=0.001, FDR=0.049) were found to have a causal relationship with the risk of DLBCL, which remained significant even after FDR correction. However, CD28- CD8br %T cell (OR=1.22, 95% CI=1.01-1.47, p=0.039, FDR=0.558), CD25hi CD45RA+ CD4 not Treg %T cell (OR=0.95, 95% CI=0.89-1.00, p=0.046, FDR=0.558), and Secreting Treg AC (OR=0.96, 95% CI=0.92-1.00, p=0.044, FDR=0.558) had p-values less than 0.05 but did not pass FDR correction, indicating a potential protective effect (**Figure 2**; **Supplementary Table S3**). In addition, IVW analysis was conducted on FL, revealing a positive correlation between CD25++ CD8br AC (OR=1.13, 95% CI=1.02-1.25, p=0.022, FDR=0.759), CD28- CD25++ CD8br %T cell (OR=1.28, 95% CI=1.01-1.62, p=0.042, FDR=0.759), CD39+ CD8br %CD8br (OR=0.90, 95% CI=0.82-0.98, p=0.016, FDR=0.759) and the risk of FL, but no causal relationship was observed (**Figure 2**; **Supplementary Table S4**).

**Figure 3:**
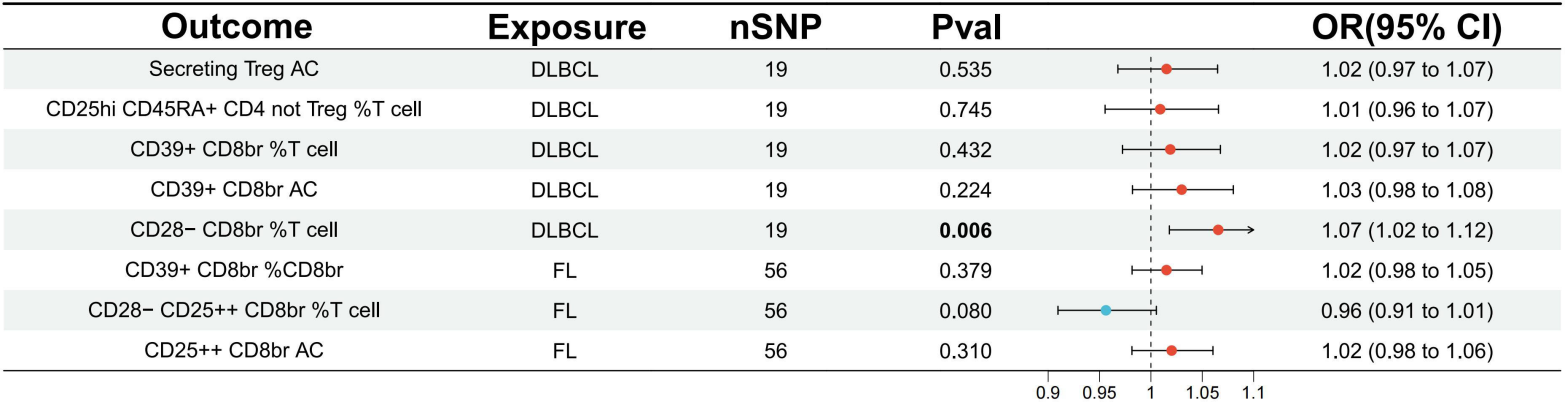
Evaluating the causal relationship between NHL and traits associated with regulatory T cell immunity using the IVW analysis method. IVW, inverse variance weighted; nSNP, the number of single nucleotide polymorphisms; DLBCL, diffuse large B-cell lymphoma; FL, follicular lymphoma.

Furthermore, a reverse analysis was conducted on Tregs immune-related traits that showed a causal relationship or potential association with DLBCL and FL. It was found that DLBCL had a causal relationship with CD28- CD8br %T cell (OR=1.07, 95% CI=1.02-1.12, p=0.006, FDR=0.032) (**Figure 3**; **Supplementary Table S5**). Sensitivity analysis did not reveal potential pleiotropy (all p for Egger intercept > 0.05), and no heterogeneity among SNPs was observed (all p for Cochran’s Q > 0.05) (**Supplementary Table S8**).

### Estimation of a causal relationship between ADs and Treg-related immune traits

**Figure 4:**
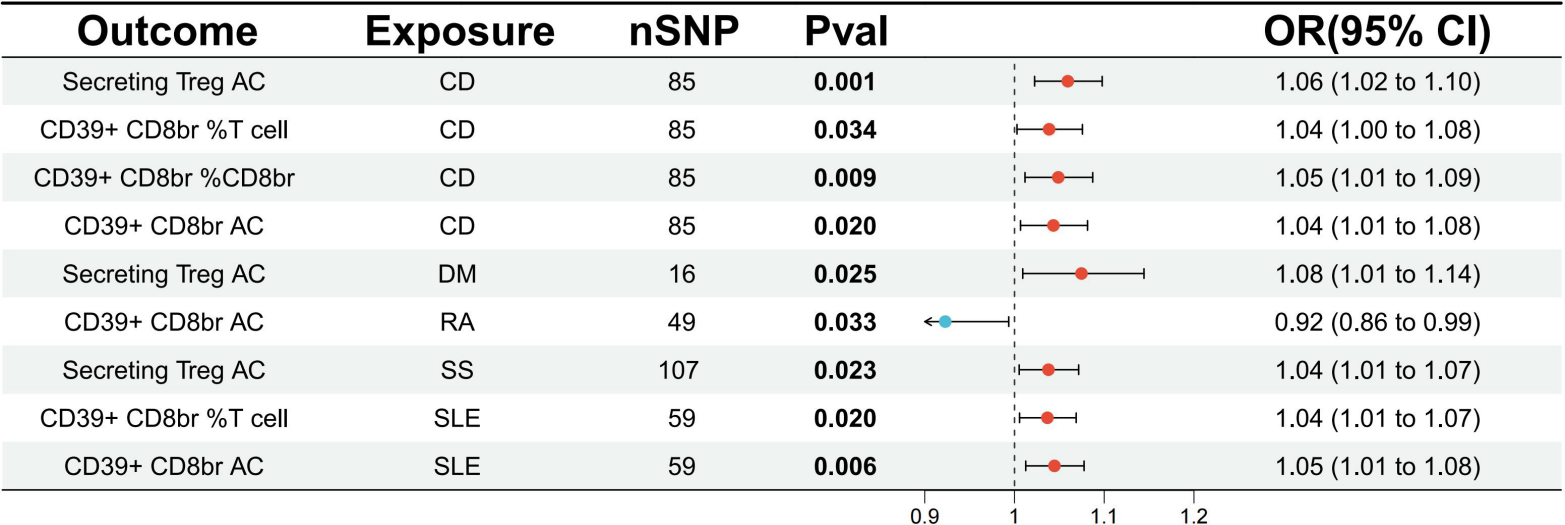
Evaluate the causal relationship between ADs and regulatory T cell immune-related characteristics based on the IVW analysis method. CD, crohn’s disease; DM, dermatopolymyositis; RA, rheumatoid arthritis; SS, sjögren’s syndrome; SLE, systemic lupus erythematosus.

To assess the impact of ADs on the Tregs immune-related traits associated with DLBCL and FL, the relevant traits were analyzed. The results showed a potential increase in the number of CD39+CD8+ Tregs subset in SLE, with CD39+ CD8br AC (OR=1.05, 95% CI=1.01-1.08, p=0.006, FDR=0.049) and CD39+ CD8br % T cell (OR=1.04, 95% CI=1.01-1.07, p=0.020, FDR=0.081) exhibiting a potential genetic association (**Figure 4**; **Supplementary Table S6**). There was a potential genetic association between CD and the CD4+ Tregs subset, as well as the CD39+ CD8+ Tregs subset. The results showed a causal relationship with Secreting Treg AC (OR=1.06, 95% CI=1.02-1.10, p=0.001, FDR=0.012), CD39+ CD8br %T cell (OR=1.04, 95% CI=1.00-1.08, p=0.034, FDR=0.069), CD39+ CD8br %CD8br (OR=1.05, 95% CI=1.01-1.09, p=0.009, FDR=0.038), and CD39+ CD8br AC (OR=1.04, 95% CI=1.01-1.08, p=0.020, FDR=0.053) (**Figure 4**; **Supplementary Table S6**). Additionally, it was found that SS may increase the number of CD4+ Tregs subset, but no causal relationship was observed, as seen in Secreting Treg AC (OR=1.04, 95% CI=1.01-1.07, p=0.023, FDR=0.182). Similar conclusions were drawn for DM, where Secreting Treg AC (OR=1.08, 95% CI=1.01-1.14, p=0.025, FDR=0.198) was identified. Moreover, RA was found to potentially decrease the number of CD39+ CD8+ Tregs subset, with CD39+ CD8br AC (OR=0.92, 95% CI=0.86-0.99, p=0.033, FDR=0.265), although it did not show a causal relationship (**Supplementary Table S6**; **Figure 4**). Heterogeneity tests revealed no heterogeneity in the above results (**Supplementary Table S8**), and in the pleiotropy tests, all outcome p-values were greater than 0.05, indicating no statistical differences. Therefore, it can be considered that there was no horizontal pleiotropy in this study, suggesting that the genetic variations investigated did not influence the outcome variables through pathways other than exposure. Additionally, a reverse analysis was conducted on Tregs immune-related traits that showed a causal relationship or potential association with ADs, revealing that the CD39+ CD8+ Tregs subset may have served as a risk factor for DM, with CD39+ CD8br AC (OR=1.17, 95% CI=1.02-1.35, p=0.024, FDR=0.193). There was a potential genetic association between the CD28-CD25+CD8+ Tregs subset and AD, with CD28- CD25++ CD8br %T cell (OR=1.08, 95% CI=1.00-1.16, p=0.045, FDR=0.362), while the remaining results were negative (**Supplementary Table S7**).

## Discussion

Previous studies have indicated that Tregs play a crucial role in controlling ADs and suppressing anti-tumor immunity. Tregs in lymphoma tissue secrete perforin and granzyme B, inducing the death of NK cells and CD8+ T cells. They can also weaken the function of CD8+ tumor-infiltrating lymphocytes (TILs) by secreting inhibitory cytokines, protecting B lymphoma cells from cytotoxic effects (23, 24). Furthermore, in certain ADs, the interaction between the inflammatory environment and immune cells may lead to abnormal proliferation and hyperfunction of Treg cells, thereby affecting the regulation of immune responses and the development of the disease (25–27). Based on a large amount of publicly available GWAS data, we, for the first time, systematically explored the risk factors for the concurrent occurrence of NHL in the context of ADs from the perspective of Tregs subset using MR method. Based on our research findings, a potential genetic association was identified between SLE and CD with the CD39+CD8+ Tregs subset. Furthermore, a potential genetic association was also established between the levels of CD39-marked CD8+ Tregs subset and the risk of DLBCL. After being validated through sensitivity tests, the above results were robust and reliable, providing new insights for the clinical predictive diagnosis and treatment of SLE or CD complicated by DLBCL.

CD8+ Tregs are primarily derived from CD8+ T cells and are rarely from the thymus. In addition to secreting immunosuppressive cytokines such as IL-10 and IL-35, they also exert immunoregulatory effects by directly inhibiting the function of effector T cells (28). Unlike CD4+ Tregs, CD8+ Tregs recognize and eliminate activated T cells with abnormal effector functions through TCR recognition of Qa-1 (29). Furthermore, CD8+ Tregs can effectively suppress the response of memory effector T cells, a function that CD4+ Tregs are unable to perform (30, 31). CD8+ Tregs can also induce tolerance through cell-cell contact with antigen-presenting cells, exerting their regulatory function. Specifically, CD8+CD28- Tregs inhibit the activation of CD4+ T cells by preventing the upregulation of costimulatory molecules in antigen-presenting cells (32). Additionally, CD8+ Tregs kill target cells through perforin, granzyme A/B, and FasL/Fas-mediated cytotoxic pathways (33). Overall, they modulate immune responses by killing target cells, secreting immunosuppressive cytokines, and inducing antigen-presenting cells’ tolerance through cell-cell contact or negative signaling.

CD8+ Tregs play an important role in autoimmune diseases by inhibiting pathological immune responses mediated by self-reactive cells and contributing to the establishment and maintenance of immune homeostasis within tissues. In ADs, CD8+ Tregs may play important roles. Li et al. identified a subset of CD8+ Tregs in autoimmune diseases that express inhibitory killer cell immunoglobulin-like receptors. These cells are more abundant in patients compared to healthy individuals and may improve disease mechanisms by killing autoreactive T cells through a negative feedback mechanism (34). Furthermore, targeting CD8+ Tregs for therapy is of significant importance in the treatment of SLE. High-dose methylprednisolone treatment can increase CD8+ CD25+ Foxp3+ Tregs, reducing disease activity in SLE patients (35). All-trans retinoic acid can increase CD4+ and CD8+ Tregs, showing potential for treating SLE and other ADs (36). Autologous hematopoietic stem cell transplantation helps to increase the levels of CD8+ Foxp3+ Tregs in the circulation of SLE patients, controlling the progression of the disease (37). Furthermore, Yao et al. found that glatiramer acetate-induced CD8 Tregs exert inhibitory activity in a Qa-1-dependent manner, relying on perforin-mediated cytotoxicity, and exhibit therapeutic effects against dextran sulfate sodium-induced colitis by targeting pathogenic CD4+ T cells (38). Therefore, targeting CD8+ Tregs may have significant implications for ADs.

Currently, research on the role of CD8+ Tregs in DLBCL is relatively limited. However, existing studies have shown that elevated levels of IL-35 and IL-10 released by CD8+ Tregs are associated with poor prognosis in DLBCL. IL-10 can induce exhaustion of CD8+ T cells, while IL-35 impairs anti-tumor immunity by cooperatively regulating the BLIMP1 receptor axis. Specifically, the expression levels of IL-35 released by CD8+ Tregs are elevated in DLBCL, particularly in the poor-prognosis activated B-cell type DLBCL subtype (39). Furthermore, elevated serum levels of IL-10 are associated with poor prognosis in NHL, especially in DLBCL (40, 41). IL-10 induces CD8+ T cell exhaustion by blocking CD28 tyrosine phosphorylation and inhibiting CD28 co-stimulatory signals. Therefore, the increased levels of IL-35 and IL-10 released by CD8+ Tregs in DLBCL may lead to compromised immune function, thereby impacting prognosis and the effectiveness of anti-tumor immunity (42).

Furthermore, the CD8+ Tregs subset is a heterogeneous group.CD39 is an ectonucleoside triphosphate diphosphohydrolase that binds to extracellular ATP (eATP) and hydrolyzes it to AMP. Another extracellular nucleotidase, CD73, further hydrolyzes AMP to adenosine (43). Adenosine, as a crucial immune regulatory factor, directly inhibits the function of NK cells, CD8+ T cells, and other immune effector cells while promoting the immune suppressive function of Tregs and myeloid-derived suppressor cells. Mandapathil et al. confirmed that adenosine generated by CD39-labeled Tregs significantly contributed to the inhibition of human T cell proliferation mediated by Tregs (44). Bertrand et al. found that CD39-labeled Tregs could effectively suppress NK cell anti-tumor immunity both *in vivo* and *in vitro* (45). Chandra et al. discovered that compared to follicular lymphoma (FL) and Burkitt lymphoma, CD39-labeled Tregs were highly expressed in DLBCL, and their upregulation was often associated with poor patient prognosis (46). Therefore, adenosine generation mediated by CD39 and CD73 plays a key role in immune regulation, impacting the functions of NK cells and CD8+ T cells, and modulating the behavior of Tregs in tumor immunity. However, research on CD39-labeled CD8 Tregs is limited at present. Parodi et al. found that blockade of CD39 significantly inhibited the suppressive activity of CD8+ Tregs (from peripheral blood and tumor microenvironment), indicating that CD39-mediated suppression is a common feature of its function (47). Zhang et al. found that the expression of CD39 in TGF-β-induced CD8 Treg cells can inhibit lupus nephritis in chronic graft-versus-host disease (cGVHD) and serve as a novel biomarker for identifying CD8+ Treg cells (48). CD39 functions in CD8+Tregs to facilitate the realization of their immunosuppressive function. In this study, we identified a subset of CD39-marked CD8+Tregs as a risk factor for the SLE or CD complicated by DLBCL. This association may be linked to the immunosuppressive role of CD39+CD8+Tregs in the tumor microenvironment, although the specific mechanisms require further investigation.

In addition, in our study, we identified a potential genetic association between the onset of SLE and CD with the elevation of CD39+CD8+Treg subset levels. Previous studies have indicated that the CD39+ CD8+ Tregs subset played a crucial role in regulating immune homeostasis, preventing excessive immune responses, and controlling autoimmune diseases by producing inhibitory cytokines and regulatory receptors (47, 49). Some studies have shown that SLE patients often have limited function or reduced numbers of Tregs (50–52). However, there is controversy regarding the quantity of Tregs in SLE research. In our study, we observed that the onset of SLE may lead to an increase in the number of CD39+CD8+ Treg subsets, and similar findings of increased numbers of other Treg subsets have also been reported 7in other studies (51, 53). This may represent an auxiliary compensatory mechanism generated by the body to maintain immune homeostasis. However, its specific mechanism needs further validation.

In this study, we first extracted effective instrumental variables based on different published GWAS large cohort data from different sources, effectively avoiding overlap between exposure and results and achieving high statistical efficacy. Second, relevant studies on ADs complicated by NHL are limited to a few case reports or clinical studies, while relevant studies on Treg-related immunophenotypes and disease are even scarcer. Previous epidemiological studies were prone to biases due to confounding factors and reverse causality. We used two-way MR analysis to determine the causal relationship between Treg-related immune traits and ADs complicated by NHL, thereby eliminating unobserved confounding factors and reverse causality and enhancing the ability to infer causality. This approach provided reliable evidence to identify a causal association between the CD39+ CD8+ Tregs subset and ADs complicated by NHL. Third, to avoid estimation bias, IVW analysis was used as the main statistical analysis method, supplemented by weighted median, MR-Egger, simple mode, and weighted mode analyses, and sensitivity analysis was performed to ensure robustness of the results. Fourth, the likely association identified in this study may contribute to further investigations of the potential mechanism of association between Treg-associated immunophenotypes and ADs complicated by NHL.

Our study inevitably had some limitations. Firstly, the proportion of Tregs cell subset in peripheral blood is usually small, thus the sample size used for FACS analysis in the original literature may have been limited, which could have introduced some bias into our analysis results (16). Furthermore, because the study is based on a European database, the conclusions cannot be extended to other ethnic groups, thereby limiting the generalizability of our results. Thirdly, when incorporating instrumental variables associated with Treg immune traits, we did not select p=5e-8 as the optimal threshold, considering the constraint imposed by the number of instrumental variables. Based on previous references (54–58), we expanded the threshold range and used p=1e-5 as the standard for screening instrumental variables. This may lead to weak efficacy of instrumental variables. Finally, further validation of our findings will be required in large-scale population-based cohort studies.

## Conclusion

There was a potential genetic association between the CD39+ CD8+ Tregs subset and the risk of DLBCL. SLE and CD were potentially genetically linked to the CD39+ CD8+ Tregs subset. Taken together, the results of this MR study suggested that the CD39+ CD8+ Tregs subset could be a potential risk factor for the concurrent occurrence of SLE or CD complicated by DLBCL. However, further large-scale prospective and in-depth mechanistic studies are needed for further verification.

## Data availability statement

The original contributions presented in the study are included in the article/**Supplementary Material**. Further inquiries can be directed to the corresponding author.

## Author contributions

QL: Writing – original draft, Writing – review & editing, Conceptualization, Data curation, Formal analysis, Funding acquisition, Investigation, Methodology, Project administration, Resources, Software, Supervision, Validation, Visualization. XZ: Writing – original draft, Writing – review & editing, Conceptualization, Data curation, Formal analysis, Funding acquisition, Investigation, Methodology, Project administration, Resources, Software, Supervision, Validation, Visualization. KL: Conceptualization, Formal analysis, Investigation, Project administration, Writing – original draft, Writing – review & editing. YW: Data curation, Methodology, Writing – review & editing. CL: Data curation, Methodology, Supervision, Conceptualization, Formal analysis, Funding acquisition, Investigation, Resources, Software, Visualization, Writing – review & editing. CG: Writing – review & editing, Conceptualization, Investigation, Software. QC: Data curation, Methodology, Supervision, Writing – original draft, Writing – review & editing. CS: Methodology, Project administration, Resources, Software, Supervision, Validation, Visualization, Writing – original draft, Writing – review & editing, Conceptualization, Data curation, Formal analysis, Funding acquisition, Investigation.

## Supplementary material

Supplementary Figure 1 Overview of biological mechanisms. **(A)** CD8+Tregs negatively regulate B cells to help control and modulate autoimmune diseases. CD8+Tregs secrete various inhibitory factors and chemokines to suppress the function of effector T cells and the activation of B cells; they express inhibitory checkpoint molecule CTLA-4 to downregulate co-stimulatory molecules CD80 and CD86; simultaneously, they upregulate inhibitory receptors such as ILT3 and ILT4 to induce tolerance and anti-inflammatory responses in DCs. **(B)** Mechanism of CD8+Tregs in tumor immune microenvironment.CD8+Tregs promote the conversion of ATP into extracellular immunosuppressive adenosine by expressing CD39 and CD73, leading to the infiltration of tolerogenic TAM and MDCS, which suppress the activity of effector T cells. CD8+Tregs also directly secrete various cytokines such as IL-10, TGF-β, and IL-2 to inhibit the tumor-killing function of effector T cells, NK cells, and DCs. Under the mediation of IDO, the interaction between CD8+Tregs and DCs converts tryptophan to kynurenine, further inhibiting the tumor-killing function of effector T cells, NK cells, and APCs. Additionally, CD8+Tregs express the inhibitory checkpoint molecule CTLA-4, which downregulates co-stimulatory molecules like CD80 and CD86.Additionally, MHC class I-restricted CD8+ Tregs are capable of killing activated T effector cells expressing Qa-1/HLA-E. Tumor cells express the CD47 anti-phagocytic signal and bind to its ligand SIRPα, inducing immune evasion in tumor cells.IL-10, Interleukin-10; TGF-β, Transforming Growth Factor Beta; CCL4, Chemokine (C-C motif) ligand 4; CTLA-4, Cytotoxic T-lymphocyte-associated protein 4; ILT3, Immunoglobulin-like transcript 3; ILT4, Immunoglobulin-like transcript 4; DC, dendritic cell;B-NHL,B-cell non-Hodgkin lymphoma.
