# Supplementary figures and images for: Exploring risk factors for autoimmune diseases complicated by non-hodgkin lymphoma through regulatory T cell immune-related traits: a Mendelian randomization study
Source: Front Immunol. 2024 May 28;15:1374938. doi: 10.3389/fimmu.2024.1374938 (PMC11165099; doi:10.3389/fimmu.2024.1374938)

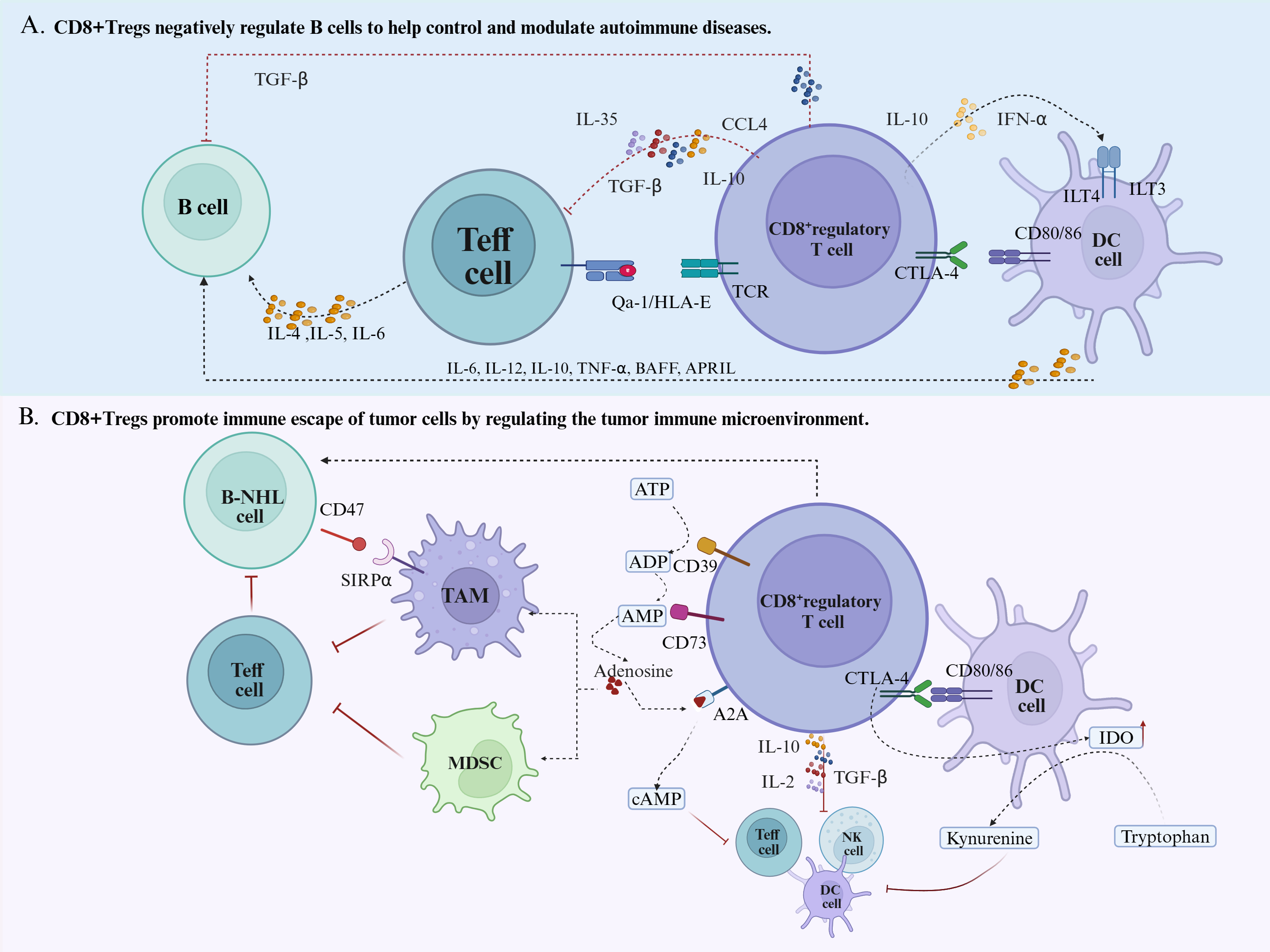

Supplement: Supplementary Figure 1 — Overview of biological mechanisms. (A) CD8+Tregs negatively regulate B cells to help control and modulate autoimmune diseases. CD8+Tregs secrete various inhibitory factors and chemokines to suppress the function of effector T cells and the activation of B cells; they express inhibitory checkpoint molecule CTLA-4 to downregulate co-stimulatory molecules CD80 and CD86; simultaneously, they upregulate inhibitory receptors such as ILT3 and ILT4 to induce tolerance and anti-inflammatory responses in DCs. (B) Mechanism of CD8+Tregs in tumor immune microenvironment.CD8+Tregs promote the conversion of ATP into extracellular immunosuppressive adenosine by expressing CD39 and CD73, leading to the infiltration of tolerogenic TAM and MDCS, which suppress the activity of effector T cells. CD8+Tregs also directly secrete various cytokines such as IL-10, TGF-β, and IL-2 to inhibit the tumor-killing function of effector T cells, NK cells, and DCs. Under the mediation of IDO, the interaction between CD8+Tregs and DCs converts tryptophan to kynurenine, further inhibiting the tumor-killing function of effector T cells, NK cells, and APCs. Additionally, CD8+Tregs express the inhibitory checkpoint molecule CTLA-4, which downregulates co-stimulatory molecules like CD80 and CD86.Additionally, MHC class I-restricted CD8+ Tregs are capable of killing activated T effector cells expressing Qa-1/HLA-E. Tumor cells express the CD47 anti-phagocytic signal and bind to its ligand SIRPα, inducing immune evasion in tumor cells.IL-10, Interleukin-10; TGF-β, Transforming Growth Factor Beta; CCL4, Chemokine (C-C motif) ligand 4; CTLA-4, Cytotoxic T-lymphocyte-associated protein 4; ILT3, Immunoglobulin-like transcript 3; ILT4, Immunoglobulin-like transcript 4; DC, dendritic cell;B-NHL,B-cell non-Hodgkin lymphoma. [file DataSheet_1.zip › Supplementary_Material/Figure S1.jpeg]
